# ﻿*Lygodium palmatum* subsp. *﻿puskariorum* (Lygodiaceae), a new subspecies from the eastern USA

**DOI:** 10.3897/phytokeys.262.165533

**Published:** 2025-08-27

**Authors:** Jordan S. Metzgar

**Affiliations:** 1 Massey Herbarium (VPI), Department of Biological Sciences, Virginia Polytechnic Institute and State University, Blacksburg, Virginia, 24061, USA Virginia Polytechnic Institute and State University Blacksburg United States of America

**Keywords:** Eastern USA, new subspecies, taxonomy, monilophytes, *
Lygodium
*

## Abstract

Lygodium palmatum (Bernh.) Sw. subsp. puskariorum Metzgar, **subsp. nov.** (Lygodiaceae) is described from the eastern USA. This taxon differs from Lygodium palmatum subsp. palmatum by its glabrous leaf undersides, absence of hairs along the fertile and sterile segment petioles, and its geographic range. It is the less widely distributed of the two subspecies.

## ﻿Introduction

**Figure 1.**
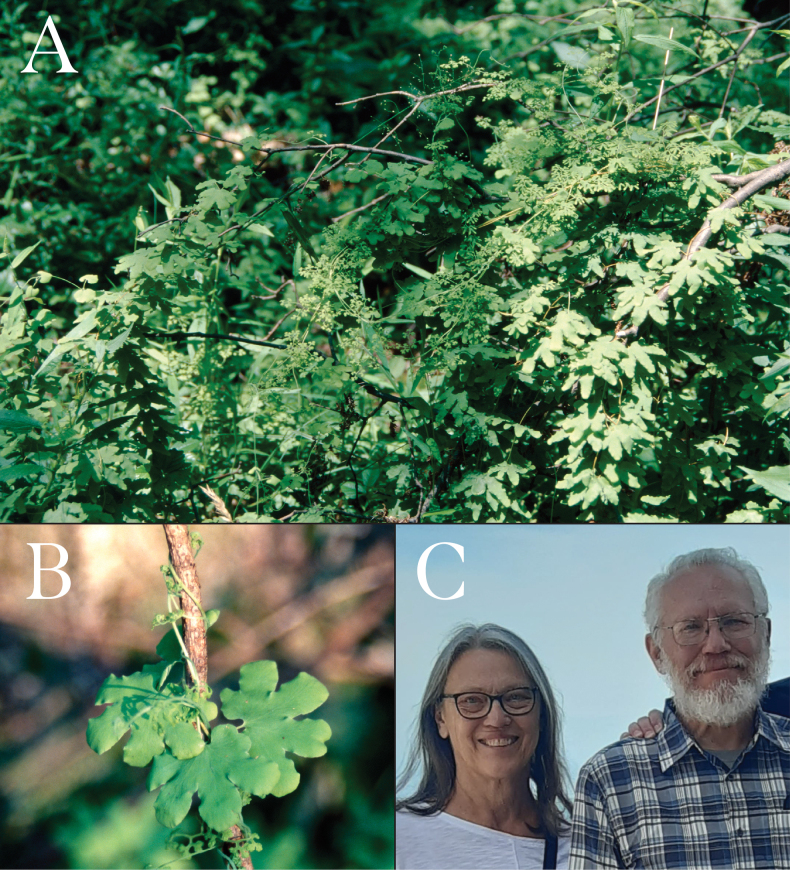
Habitat, habit, and Puskars. A. Lygodium palmatum subsp. puskariorum grows in sunny gaps in moist acidic woods; B. Lygodium palmatum subsp. puskariorum has a twining habit with palmately lobed sterile leaflets and reduced fertile leaflets; C. Donna and Allan Puskar are the namesakes for the new taxon Lygodium palmatum subsp. puskariorum. Photograph by Matthew Puskar.

The Hartford fern (*Lygodium palmatum* (Bernh.) Sw.) is one of eastern North America’s most distinctive and culturally resonant ferns. This fern is the only native *Lygodium* species in both the region and the United States overall, and it is also the region’s only native twining fern (Shaver 1954; Cobb 1956; Fig. 1A, B). Despite its unique morphology, the Hartford fern was not described until 1801. The species is endemic to the eastern half of the USA, stretching from New England to Georgia and west to Kentucky, with disjunct populations in Michigan.

The species was heavily collected in the late 1800s during the pteridomania craze (Eaton 1879). As a result, the Connecticut legislature banned its harvest in 1869. This act seems to have been a regulatory milestone and was described shortly after as “probably…the only instance in statute law where a plant has received special legal protection solely on account of its beauty” (Eaton 1879). The Hartford fern also received acclaim from prominent naturalist and author Henry David Thoreau, who discovered a population near his famed Walden Pond abode. The population’s location was lost for decades until its rediscovery in 1978 (Angelo 1985).

Molecular analyses have consistently retrieved *L. palmatum* in a small clade that is sister to the remaining species in the genus (Wikström et al. 2002). The species is closely related to the New Caledonian *L. hians* E. Fourn. (Pelosi et al. 2024) and also to the southwestern Pacific *L. articulatum* A. Rich. (Madeira et al. 2008; Pelosi et al. 2024).

Authors have long noted variation in pubescence of the leaflet undersides (Eaton 1879; Shaver 1954; Brown 1984; Nauman 1993). Garrison (1992, 1998) was the first to note a geographic pattern in the distribution of pubescent specimens, concluding that the state of Maryland was a geographic divide between pubescent and glabrous forms. Specimens collected to the south or west of Maryland were generally pubescent, and specimens collected north or east of Maryland were generally glabrous (Garrison Hanks 1998). The unique morphology of *L. palmatum* and consequent ease of its identification may have caused this intraspecific variation to be neglected (Garrison 1992). Later works have continued to note the existence of this pattern without drawing conclusions about its taxonomic significance and instead highlighting the need for detailed research on this variation (e.g., Weakley et al. 2012). I address this need in the present study.

## ﻿Methods

I characterized the presence and distribution of leaflet pubescence in *Lygodium palmatum* using an extensive study of herbarium material. More than 300 herbarium specimens were inspected from eight different herbaria (B, BHO, CM, DUKE, GMUF, LFCC, NCU, and VPI). These herbaria were selected based on their sizable holdings representing geographic regions where the two morphotypes might overlap or regions containing disjunct populations. Specimens were examined using a dissecting microscope with magnification from 20–50×. Specimen collection locations were georeferenced as needed and were mapped along with environmental traits using QGIS software (QGIS Development Team 2024).

**Figure 2.**
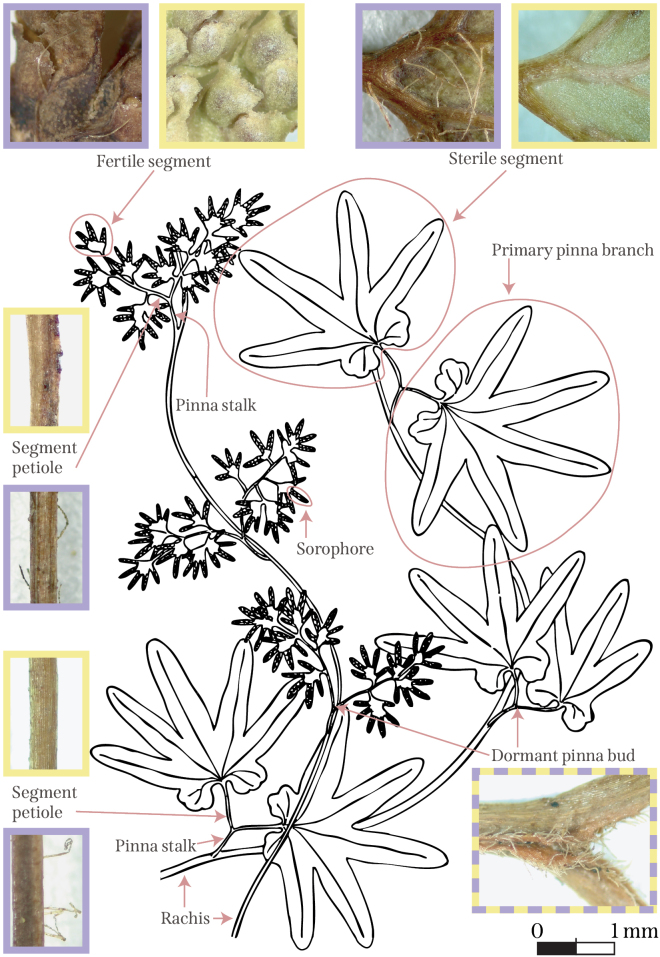
Illustrated morphological terminology. Both subspecies of *Lygodium palmatum* have twining fronds with complex dissection patterns. The main identification characters are illustrated with photomicrographs taken at 20× magnification, with the character state for Lygodium palmatum subsp. puskariorum outlined in light purple and the character state for Lygodium palmatum subsp. palmatum outlined in yellow. The dormant pinna bud character is invariant between subspecies and is outlined in both light purple and yellow. All photomicrographs for L. palmatum subsp. puskariorum are from a single representative specimen (Flentje 7; VPI 112295). All photomicrographs for L. palmatum subsp. palmatum are from one representative Massachusetts specimen (Ahles 78547; VPI 57060). The line drawing is modified from Ruth Sinclair George’s illustration (Small 1938).

These samples were scored for the presence of hairs on the sterile leaflet undersides, sterile leaflet segment petioles, fertile leaflet undersides, and fertile leaflet segment petioles. The leaf morphology of *Lygodium palmatum* is complex, and conventional fern morphology terminology is not readily transferable (Garrison Hanks 1998). I followed the suggested terminology proposed by Garrison Hanks (1998) to describe *Lygodium* sporophyte morphology (Fig. 2).

A single leaf has leaflets that are alternately arranged along the rachis. These leaflets are divided with a complex geometry. Each sterile leaflet has a short pinna stalk that divides into two segment petioles, each terminating in a single palmately lobed segment. A dormant pinna bud is produced at the division of the pinna stalk. The fertile leaflets have a similar morphology, except they have a winged segment petiole with multiple segments. Indusiate sporangia are arranged in two rows on finger-like sorophores (Fig. 2; Garrison Hanks 1998).

## ﻿Results and discussion

The presence or absence of hairs on the abaxial leaf surfaces can be used to reliably distinguish the glabrous and pubescent morphotypes of *Lygodium palmatum*. Specifically, the pubescent morphotype has sterile leaflet undersides with white to reddish-orange hairs that are 0.8–2.0 mm long (Fig. 2). These hairs typically extend onto the sterile leaflet segment stalk. A small number of individuals of the pubescent morphotype are sparsely hairy.

The fertile leaflets are more difficult to examine due to the reduced amount of lamina and the presence of indusiate sori. However, specimens of the pubescent morphotype usually possess at least some hairs on the abaxial surface of their fertile leaflets and on the fertile leaflet stalks (Fig. 2). Additionally, the glabrous morphotype will occasionally produce a hair on the fertile leaflet tissue, making identification using only fertile tissue more challenging.

The unwitting examination of a dormant bud at the primary branch apex can also confound identification (Fig. 2). Both morphotypes can display dormant buds that are covered in similar hairs or buds that are glabrous. Characteristic examples of each of these leaf characters are shown for both the glabrous and pubescent morphotypes (Fig. 2).

**Figure 3.**
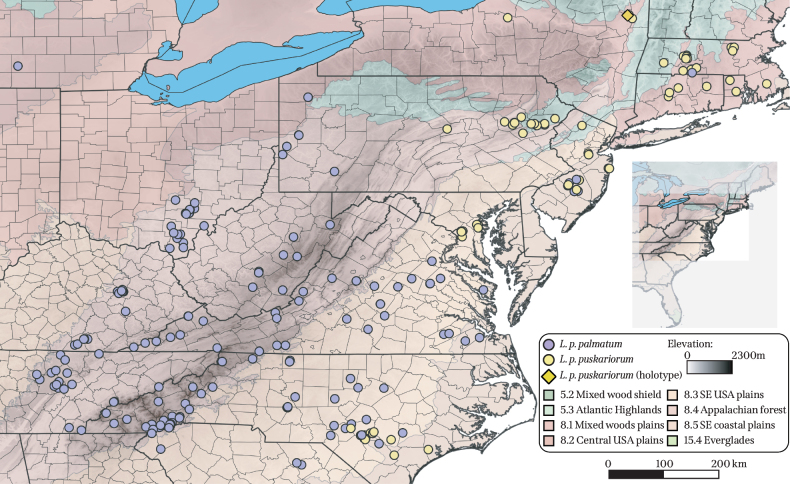
Distribution map. Populations of Lygodium palmatum subsp. puskariorum are depicted with yellow circles, and the holotype is indicated with a dark yellow diamond. Populations of Lygodium palmatum subsp. palmatum are depicted with purple circles. The provenance of the Lygodium palmatum subsp. palmatum holotype (Muhlenberg s.n.; Herb. Willd. 19484; B) is unknown, so it is not depicted on the map. Ecoregions are portrayed in tones of green and red. Dark shading indicates elevation.

I examined 302 herbarium specimens and found that 95 specimens represent the glabrous morphotype and 207 specimens represent the pubescent morphotype, including the original material for *Lygodium palmatum* (B, Herb. Willd. 19484, Muhlenberg s.n., both sheets, photo! photomicrographs!). Specimens are listed and organized by state in the “Specimens examined” section. The morphotypes display a strong geographic pattern, with the glabrous morphotype concentrated in the northeastern and mid-Atlantic USA. The pubescent morphotype is found in the midwestern USA, the mountainous southeastern USA, and the eastern coastal plain (Fig. 3).

Ecoregions can serve as strong boundaries impacting species–and community-level biodiversity (Smith et al. 2018) and can be used to broadly characterize both *L. palmatum* morphotypes. The morphotypes are broadly separated by ecoregion, with a narrow coastal overlap zone (Fig. 3). Most populations of the glabrous morphotype occur in the Atlantic highlands and mixed woods plains (ecoregions 5.3 and 8.1, respectively), while most populations of the pubescent morphotype are found in the Appalachian forests ecoregion (ecoregion 8.4). A minority of populations of both morphotypes are found in the southeastern USA plains and southeastern USA coastal plains (ecoregions 8.3 and 8.5, respectively). The overall distribution patterns of the morphotypes correspond to distinct Level II ecoregions, suggesting ecological divergence possibly linked to edaphic and climatic factors. Areas of the southeastern USA coastal plains and southeastern USA plains contain the main contact zones between the two morphotypes. In particular, southern New Jersey, southeastern North Carolina, and central Massachusetts represent areas of secondary contact (Fig. 3).

Leaf pubescence characters are commonly used in plant taxonomy and have been sufficient, in certain cases, to define species or infraspecific taxa on their own. Sympatric morphotypes distinguished by pubescent versus glabrous leaves have even been recognized as full species. *Cyclodium pubescens* Bohn & Labiak overlaps geographically almost completely with its close relative *C. meniscoides* (Willd.) C. Presl but was distinguished from that taxon by the presence of “abundant, erect hairs” on its laminar tissue (Bohn et al. 2020). Similarly, allopatric taxa from the same floristic regions have been afforded specific status based on leaf hair characters. The neotropical fern *Hypolepis rigescens* (Kunze) T. Moore is allopatric with respect to *H. tenerrima* Maxon and is distinguished by the presence of glandular rather than needle-shaped hairs (Schwartsburd and Prado 2016). Among Pacific filmy ferns, *Hymenophyllum paniense* Ebihara & K. Iwats. was described as a species due to distinct leaf hair traits (Ebihara et al. 2003). *Hymenophyllum pilosissimum* C. Chr. was similarly recognized as distinct from *H. obtusum* (Hook. & Am.) Copel. based on leaf hair branching patterns (Moore et al. 2010). The subspecific ranks of subspecies and variety are also used to recognize morphotypes distinguished by leaf hair traits. Within *Pteridium aquilinum* (L.) Kuhn, three of the recognized subspecies have overlapping ranges and are distinguished by leaf pubescence characteristics (P. aquilinum ssp. latiusculum (Desv.) Hultén, P. aquilinum ssp. pseudocaudatum (Clute) Hultén, and P. aquilinum ssp. pubescens (Underw.) J.A. Thomson, Mickel & Mehltr.; Thomson et al. 2008). The rank of variety is also employed for classifying sympatric morphotypes distinguished by the presence or absence of pubescence; for example, Coniogramme intermedia Hieron. var. glabra Ching and Coniogramme intermedia var. intermedia (Zhang and Ranker 2013), as well as Diplazium esculentum (Retz.) Sw. var. pubescens (Link) Tardieu & C. Chr. and Diplazium esculentum var. esculentum (He and Kato 2013).

**Figure 4.**
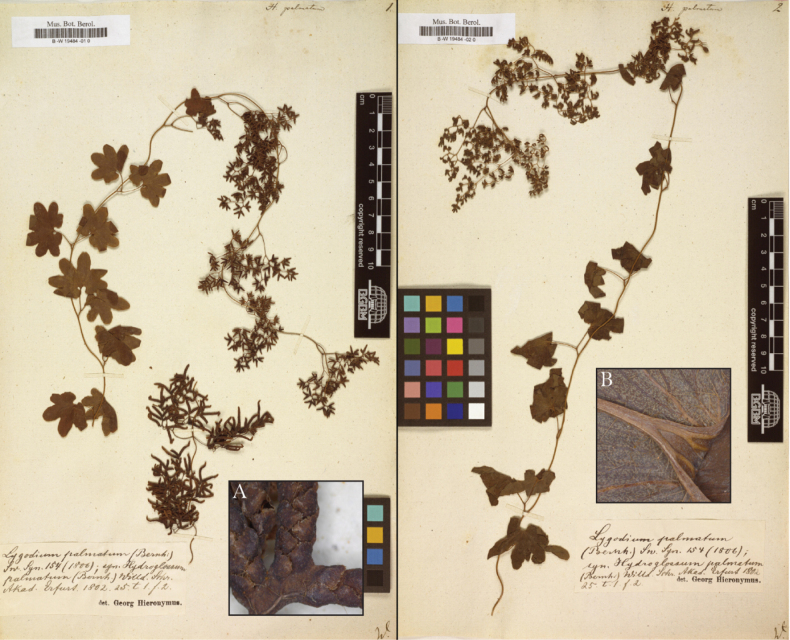
Holotype. The holotype of Lygodium palmatum subsp. palmatum (Muhlenberg s.n.; Herb. Willd. 19484; B; Curators Herbarium B 2025). Insets: photomicrographs documenting abaxial pubescence on A. A fertile leaflet and B. A sterile leaflet. Photomicrographs courtesy of Juraj Paule (Curators Herbarium B 2025).

The glabrous and pubescent morphotypes within *Lygodium palmatum* represent subspecific variation that is geographically patterned and reliably distinguishable using morphology. This population-level differentiation likely represents incipient speciation and warrants the recognition of separate subspecies (Yatskievych and Moran 1989). The holotype for *L. palmatum* (Muhlenberg s.n.; Herb. Willd. 19484; B; Fig. 4) is pubescent, so the glabrous morphotype should be recognized as a new subspecies.

### ﻿Taxonomic treatment

#### ﻿ Lygodium palmatum subsp. puskariorum

Taxon classificationPlantaeSchizaealesLygodiaceae

Metzgar subsp. nov.

D8B17744-025B-546B-A9DD-D8633393C330

urn:lsid:ipni.org:names:77368258-1

Figs 1 , 2 , 3 , 5

##### Type.

United States. New York • Saratoga, Northumberland Township, 147 Gurnspring Road, in opening behind houses on east side of road, 26 July 2003, *J. Metzgar 44* (holotype: DUKE! 397033).

**Figure 5.**
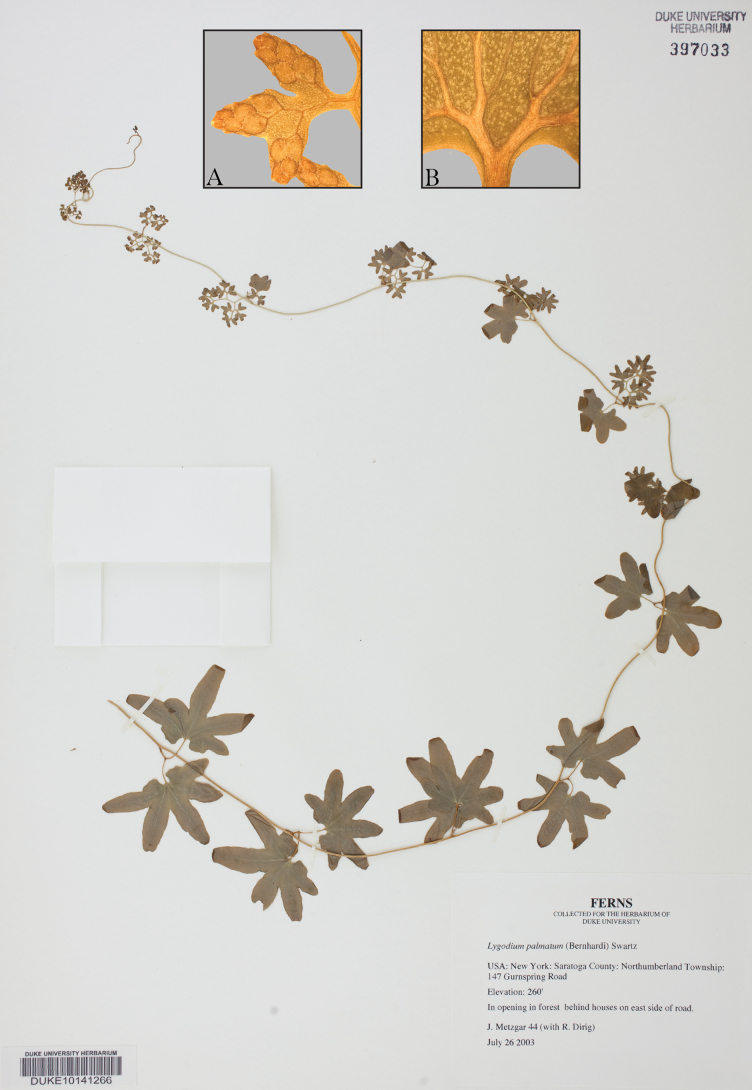
Holotype. The holotype of Lygodium palmatum subsp. puskariorum (Metzgar 44; DUKE 397033). Insets: A. Photomicrograph of a glabrous fertile leaf underside; B. Photomicrograph of a glabrous sterile leaf underside.

##### Diagnosis.

Lygodium palmatum subsp. puskariorum differs from the similar Lygodium palmatum subsp. palmatum by the absence of white to reddish-orange hairs on the abaxial surface of the sterile leaflets, the sterile leaflet segment petioles, the abaxial surface of the fertile leaflets, and the fertile leaflet segment petioles.

##### Description.

Roots with white hairs. Rhizome approximately 1 mm wide and subterranean with dark red hairs. Leaves are evergreen and grow to three meters in length with a thin, twining stipe. Compound pinnae alternately arranged with a pinna stalk borne from the rachis. The pinna stalk branches into two primary pinna branches. Dormant pinna bud that can be glabrous or pubescent found at this branching junction. Each sterile primary pinna branch has a segment petiole terminating in a sterile segment (pinnules). Sterile segments palmately lobed, with 4–6 lobes per segment. Segment petioles and the abaxial surface of sterile segments are glabrous. Fertile segments are dimorphic with respect to the sterile segments. Fertile segments have reduced lamina with sporangia borne on projecting sorophores. The abaxial surface of the fertile segment lamina and the fertile segment petiole lack hairs. Sporangia are indusiate and occur in 4–10 pairs on a sorophore. Spores mature in the fall or early winter.

##### Etymology.

The epithet “puskariorum” is in honor of both Allan and Donna Puskar (Fig. 1C), retired school teachers from Wellsboro, PA. Their family name is pronounced “push-car.” Allan and Donna taught my high school ecology and biology courses, fueling my love for science and natural history. They have always possessed a deep commitment to their students, with a special compassion for me and many other students who did not quite belong, were down on their luck, or yearned for a larger world. They are also lifelong fern enthusiasts who, one fateful summer in 2001, taught me the basics of fern identification and inadvertently launched my career as a pteridologist. *Lygodium palmatum* has long been a favorite fern of theirs.

##### Common name.

I suggest “Puskar’s climbing fern” and “Puskar’s Hartford fern” as common names for L. palmatum subsp. puskariorum and suggest “southern climbing fern” and “southern Hartford fern” as common names for L. palmatum subsp. palmatum.

##### Geographic distribution and habitat.

Lygodium palmatum subsp. puskariorum is distributed across the northeastern and mid-Atlantic USA, being found in Connecticut, Maryland, Massachusetts, New Jersey, New York, North Carolina, Pennsylvania, and Rhode Island (Fig. 3). Southern New Jersey, southeastern North Carolina, and central Massachusetts represent areas of secondary contact with L. palmatum subsp. palmatum. The Puskar’s climbing fern grows in moist, acidic soil in forests, bogs, and roadsides.

##### Conservation assessment.

*Lygodium palmatum* sensu lato has a global conservation ranking of G4 (“apparently secure”; Weakley 2025). However, most states in L. palmatum subsp. puskariorum’s range already list *L. palmatum* s.l. as Critically Imperiled (S1), Imperiled (S2), or Vulnerable (S3; NatureServe 2025). Additionally, the taxon is sensitive to development and generally does not survive transplantation or cultivation (Montgomery 1989). I recommend Imperiled status (G2) for L. palmatum subsp. puskariorum due to its more narrow distribution and scarcity throughout most of its range.

##### Additional specimens examined.

**United States. Connecticut** • Hartford, Plainville, Sep. 1877, J. Bishop s.n. (CM) • Hartford, probably from vicinity of Hartford, Ziegler s.n. (CM) • Hartford, Southington, s. coll. s.n. (CM); no further locality, H. Denslow s.n. (DUKE) • Plainville, Aug 1883, A. Seymour 134 (DUKE); **Maryland** • Anne Arundel, along Severn River near Benfields, 18 Aug 1966, L. Henry s.n. (CM) • Anne Arundel, North side of Severn Run, 19 June 1981, M. Windham 81-26 (DUKE) • Charles, Sevier River, 18 Aug. 1960, J. Benedict 6432 (NCU, VPI) • Prince Georges, Bladensburg, 26 Mar. 1910, H. Bartlett 1887 (DUKE, NCU) • Prince Georges, Hyattsville, Nov. 1899, C. Pollard, Louis s.n. (NCU) • Prince Georges, Suitland, 9 Sept. 1899, E. Steele (DUKE); **Massachusetts** • Hampden, Palmer, 25 Aug 1925, G. Bennett G163 (DUKE) • Hampden, Springfield, 31 Aug. 1934, F. Sargent (DUKE) • Hampden, Wilbraham, 11 Sept. 1935, F. Seymour 4406 (DUKE) • Hampshire, Amherst, 11 July 1980, C. Hellquist 14577 (CM) • Hampshire, Amherst, 28 June 1928, S. Ewer s.n. (NCU) • Hampshire, Amherst, 4 Nov. 1889, G. Nash 99 (CM) • Hampshire, Amherst, S. Estabrook s.n. (NCU) • Hampshire, East Hampton, 29 June 1872, E. Miller s.n. (CM) • Hampshire, North Amherst, 23 Aug. 1895, A. Beals 37 (DUKE) • Hampshire, North Hadley, 29 Nov. 1922, R. Sanborn 2082 (DUKE) • Middlesex, Ayer Junction, July 1883, S. Steele (DUKE) • Middlesex, Shirley, F. Sargent (DUKE) • Middlesex, Still River, Oct. 1910, R. Ware 766 (NCU) • no further locality, Canby (CM) • no further locality, H. Mann s.n. (CM) • no further locality, s. coll. s.n. (NCU) • Plymouth, Lakeville, 12 Sept. 1986, B. Sorrie 3652 (NCU); **New Jersey** • Burlington, 1 mi NNE of Taunton, 10 Aug. 1926, W. Benner s.n. (CM) • Burlington, Hampton Furnace, 3 July 1922, G. Bassett (DUKE) • Burlington, near New Lisbon, 11 June 1950, D. Krouse s.n. (CM) • Middlesex, Milltown, 25 Dec. 1924, G. Johnson (DUKE) • Middlesex, New Brunswick, 1899, J.Blodgett s.n. (CM) • Monmouth, Shark River, 5 Oct. 1855, F. Hexamer s.n. (CM) • Sussex, Pine Swamp, 1838, Wolle Herbarium s.n. (CM); **New York** • Onondaga, Cicero Swamp, near Syracuse, 22 Nov. 1966, M. Faust s.n. (CM) • Saratoga, near Gansevoort, 30 Oct. 1938, H. House 26344 (NCU) • Saratoga, Town of Northumberland, Oct. 1938, O. Phelps s.n. (NCU); **North Carolina** • Bladen, 1 June 1979, R. Rebertus s.n. (NCU) • Cumberland, 1 mile east of Stedman, 26 Dec. 1932, H. Totten s.n. (NCU) • Cumberland, 1 mile southeast of Stedman, 26 May 1961, A. Radford 43676 (NCU) • Cumberland, 7 miles south of Fayetteville, 17 Oct. 1931, M. Smyth s.n. (NCU) • Cumberland, About 1 mile southwest of Stedman, 26 Nov. 1964, H. Totten 1832 (NCU) • Cumberland, Along bank of Rock Fish Creek, 8 Oct. 1939, A. Radford s.n. (NCU) • Cumberland, Along bank of Rockfish Creek, 8 Oct. 1939, L. Stewart s.n. (DUKE) • Cumberland, Along Rockfish Creek, 8 May 1938, D. Correll 9052 (DUKE) • Cumberland, Ardlussa, 1 Oct. 1931, M. Vardell s.n. (DUKE) • Cumberland, Dunns Creek, 16 Oct. 1982, L. Raubeson 399 (NCU) • Cumberland, Fayetteville, 23 Sept. 1934, J. Benedict 3078 (VPI) • Cumberland, North side of Rockfish Creek, 28 Oct. 1969, S. Leonard 2804 (NCU) • Cumberland, Rock Fish Creek, 15 March 1964, T. Creem 316 (NCU) • Cumberland, Vicinity of Fayetteville, 1 May 1937, R. Clark (DUKE) • Onslow, East side of Sandy Run Swamp, 8 Aug. 1997, M. Strong 1571 (NCU) • Sampson, 6 miles south of Clinton, 27 Mar. 1938, L. Anderson 6159 (DUKE); **Pennsylvania** • Carbon, Mauch Chunk, Oct. 1873, C. Shaefer 11191 (CM) • in Poconos, 17 July 1970, E. Laport s.n. (NCU) • Luzerne, Hells Kitchen, Nescopeck Creek, 19 Sept. 2022, R. Sim s.n. (CM) • Luzerne, Nescopeck State Park, 10 July 2004, J. Metzgar 87 (DUKE) • Luzerne, Ten Mile Run, 1 Oct. 1938, T. Darling (VPI) • Monroe, near Tannersville, July 1874, S. Knipe s.n. (CM) • Monroe, Tunkhannock Township, 6 Sept. 1936, F. Trembley 603 (DUKE) • Monroe, Tunkhannock Township, 9 Sept. 1936, F. Trembley s.n. (NCU) • Monroe, Tunkhannock Twp, 8 July 2015, W. Gleason F15GLE35-E (CM) • Pike, Pocono Mountain, Turners Swamp, July 1874, S. Knipe s.n. (CM); **Rhode Island** • Kent, Anthony, 18 Sept. 1886, J. Collins s.n. (NCU) • Kent, Anthony, 26 Sept. 1885, G. Leland s.n. (CM) • Kent, Coventry, 16 Oct. 2020, J. Collins s.n. (NCU).

##### Representative specimens examined of *Lygodium palmatum*

**(Bernh.) Sw. subsp. palmatum. United States. Kentucky** • Breathitt, Near Morris Fork, S. VanderMeer s.n. (NCU) • Harlan, Above US 421, about 1 mile from Virginia line, 17 Feb. 1965, M. Smyth 508 (VPI) • McCreary, Cumberland Falls State Park, 15 Sept. 1982, F. Utech 82–462 (CM) • Powell, Trail to Rock Bridge Natural Bridge Park, 14 Apr. 1958, C. Gunn K30 (NCU) • Whitley, Cumberland Falls State Park, 24 Aug. 1961, L. Henry s.n. (CM) • Wolfe, 12 Sept. 1969, P. Higgins s.n. (NCU); **Massachusetts** • Hampden, Hampden, Oct. 1913, S. Bliss s.n. (NCU); **Michigan** • Kalamazoo, S of Kalamazoo, 14 Oct. 1974, R. Simpson s.n. (LFCC); **New Jersey** • Burlington, 1 mi NNE of Taunton, 10 Aug. 1926, W. Benner s.n. (NCU); **North Carolina** • Alleghany, 9 Sept. 2012, D. Goldman 4220 (NCU) • Ashe, Long Hope Creek Valley, 11 Oct. 1969, J. Moore 2799 (NCU) • Bladen, 1.6 mi ESE of Ammon, 14 July 1999, B. Sorrie 10217 (NCU) • Buncombe, 2 miles southwest of Black Mountains, 29 Aug. 1948, E. Browne s.n. (NCU) • Burke, 3 mi N of Optimist Park, 3 Apr. 1971, R. Harbison Pharr 1013 (NCU) • Cabarrus, Cabarrus, 16 Aug. 1979, D. Wickland 2126 (NCU) • Chatham, In ore mine at Mt. Vernon Springs, 13 May 1953, C. Lindley s.n. (NCU) • Cherokee, Above Granny Squirrel Gap, 17 July 2002, C. McCartney s.n. (NCU) • Cumberland, Rockfish Creek, 7 Aug. 1957, H. Ahles 33420 (NCU) • Davidson, near Silver Hill, 8 May 1988, R. Wilbur 54883 (DUKE) • Durham, NC Gamelands Trail, 30 Aug. 1992, R. Wilbur 61438 (DUKE) • Graham, approx. 3.5 mi I from turnoff to Joyce Kilmer Memorial Forest, 15 Sept. 2000, C. McCartney s.n. (NCU) • Harnett, west of Stewarts Creek, 25 Aug. 2005, B. Sorrie 11702 (NCU) • Henderson, at Pine Knob (or Brickyard) Mine, 10 Sept. 1978, D. Wickland 2452 (NCU) • Hoke, Fort Bragg, 16 Dec. 1992, B. Sorrie 7117 (NCU) • Jackson, headwaters of Tuckaseigee River, 28 June 1970, S. Leonard 3315 (NCU) • Macon, near Snow Hill, March 1956, M. Burgess s.n. (NCU) • Madison, Upper Shutin Creek Road, 6 June 1956, O. Freeman 56314 (NCU) • McDowell, Approximately 5 miles north of Marion, 5 Apr. 1980, S. Bentley 2 (VPI) • Mitchell, near Spruce Pine, summer 1950, E. Frees s.n. (DUKE) • Moore, 1.5 mi SE of Pinehurst, 16 Sept. 1931, R. Wicker s.n. (NCU) • Mountains of N. Carolina, Sept. 1876, G. Engelmann s.n. (NCU) • Orange, South side of Eno Mountain Road, 24 Aug. 2005, L. Giencke 2 (NCU) • Rowan, Gold Hill Mine, 3 Oct. 1979, D. Wickland 2447 (NCU) • Scotland, Sandhills Game Lane, 20 Oct. 1991, B. Sorrie 6040 (NCU) • Stokes, Above Lower Cascades, 28 May 1937, H. Blonquist 9299 (DUKE) • Transylvania, Brevard, 1 Aug. 1933, H. Thackston s.n. (DUKE) • Wake, 3 Mar. 1990, J. Perry 90-2 (NCU); **Ohio** • Athens, Carbondale Forest, 29 May 1939, F. Bartley s.n. (BHO) • Gallia, Greenfield Twp., 20 June 1991, G. Rhinehalt 257 (BHO) • Hocking, 4 Sept. 1938, W. Porter 10A (BHO) • Jackson, 1–1/2 mi. south of Jackson, May 1961, A. Blickle s.n. (BHO) • Lawrence, Mason Twp., 26 July 1993, M. Ortt 3509 (BHO) • Meigs, Salisbury Twp., 27 July 1994, G. Rhinehalt 949 (BHO) • Vinton, Coalmont Hollow, 12 July 1956, G. Hall 1861 (BHO); **Pennsylvania** • No further locality, *Muhlenberg s.n.* (B, Herb. Willd. 19484, photo! photomicrographs!) • Crawford, 1 mi N of Sugar Lake, 4 Aug. 1959, L. Henry s.n. (CM) • Washington, 3.2 km NNW of Florence, 1997, M. Bowers s.n. (CM) • Westmoreland, Acme, 29 Sept. 1973, R. Little s.n. (CM); **South Carolina** • Anderson, Clemson South Woods, 9 Nov. 1971, B. Snyder 1 (NCU) • Darlington, Hartsville, 20 Apr. 1932, B. Smith s.n. (NCU) • Greenville, tributary of Matthews Creek, 13 May 1972, G. McDowell s.n. (NCU) • Oconee, 1.6 mi N of Jocassee, 6 Dec. 1987, S. Hill 19053 (CM); **Tennessee** • Blount, Laurel Creek Bog Vicinity: Cades Cove, 10 July 1936, W. Barksdale s.n. (DUKE, NCU) • Campbell, Morley, 18 May 2023, J. Bright s.n. (CM) • Cumberland, junction of Dady’s and Bird Creeks, 30 Aug. 1939, S. Dyal 1323 (NCU) • Fentress, Glade Branch, 22 Sept. 1957, H. Rock 1000 (DUKE, NCU) • Morgan, between Glades and Grimsley, 28 Sept. 1966, K. Rogers 40559 (NCU) • Polk, Ocoee River Gorge, 29 Sept. 2010, J. Shaw s.n. (NCU) • Putnam, east of Monterey, 18 Sept. 1951, s. coll. s.n. (NCU) • Roane, along Piney Creek • 1 Sept. 1937, D. Correll 8120 (DUKE) • Scott, 12 mi WSW of Oneida, 22 Aug. 1987, S. Thompson 4201 (CM); **Virginia** • Alleghany, Fan Mts., 28 June 1988, C. Stevens 20372 (VPI) • Augusta, South of Stuarts Draft, 7 Sept. 1935, L. Carr (VPI) • Buckingham, 21 Apr. 1905, H. Robeson (VPI) • Campbell, just beyond Fountain Hotel, 12 Jan. 1967, R. Freer 4777 (NCU) • Caroline, east of Peutrock, 9 July 1969, A. Harvill 21866 (GMUF) • Craig, Sweet Springs Turnpike, Barbours Creek Wilderness, E. Flentje 7 (VPI) • Dickenson, Breaks Interstate Park, 26 Aug. 1982, T. Wieboldt 4509 (VPI) • Fluvanna, 1.4 mi. NE of Wilmington, 9 Sept. 1998, G. Fleming 14748 (GMUF) • Giles, Along Little Stony Creek, 29 Apr. 1944, C. Handley 404 (VPI) • Grayson, 5 miles south of Galax, 1 May 1991, T. Wieboldt 7580 (VPI) • Hanover, 1.5 mi. NE of Hewlett, 24 Aug. 1996, G. Fleming 12270 (GMUF) • Hanover, 2.5 km northeast of Hewlett on St. Rt. 601, 24 Aug. 1996, T. Wieboldt 9552 (VPI) • Highland, Laurel Fork, 11 Oct. 1969, C. Stevens 1550 (VPI) • Isle of Wight, Zuni-Walters Road, 26 Nov. 1977, D. Eggers Ware 6929 (NCU) • Lancaster, Belwood Swamp, 30 Sept. 1973, Stanley (GMUF) • Lee, 1 mile northwest of Rose Hill, 4 Sept. 1977, T. Wieboldt 3164 (VPI) • Louisa, Near Mineral, 14 Oct. 1992, C. Stevens 23294 (VPI) • Montgomery, W of Blacksburg, 14 Sept. 1993, T. Wieboldt 8820 (VPI) • Patrick, Meadows of Dan, 1 June 2006, R. Lutts (VPI) • Prince Edward, Farmville, 1 Oct. 1935, E. Stevens (VPI) • Rockbridge, west of Bells Valley, 14 Sept. 2021, J. Townsend 7109 (VPI) • Suffolk City, ODU Research Lab, 17 Sept. 1980, L. Musselman 6081 (NCU) • Surry, near Homeville, 24 May 1939, A. Massey 3022 (NCU, VPI) • Sussex, 2 miles N of Sussex, 21 July 1968, A. Harvill 19864 (GMUF) • Wise, 4 miles south of Norton, VA, 7 Aug. 1985, T. Wieboldt 5797 (VPI) • Wise, Oakwood, 31 May 2013, R. Wright 11207 (VPI); **West Virginia** • Fayette, Laurel Creek, 30 May 1973, C. Baer s.n. (NCU) • Greenbrier, Lewisburg, Aug. 1922, F. Gray 36 (CM) • Nicholas, along Meadow River, 24 Aug. 1946, W. Legg 601 (CM, NCU) • Randolph, Monongahela National Forest, 1 June 2022, B. Streets 6231 (NCU) • Tucker, near Davis, 8 Sept. 1975, E. Ruffing s.n. (CM) • Upshur,1/2 mile southwest of Indian Camp, 8 July 1938, J. Benedict 4538 (VPI) • Webster, no further locality, 11 Aug. 1940, M. Cunningham 3589 (CM).

## Supplementary Material

XML Treatment for Lygodium palmatum subsp. puskariorum

## ﻿Additional information

### Use of AI

No use of AI was reported.

### Author ORCIDs

Jordan S. Metzgar https://orcid.org/0000-0002-4641-9929

### Data availability

All of the data that support the findings of this study are available in the main text.
