# Corrigendum: Metzgar JS (2025) Lygodium palmatum subsp. puskariorum (Lygodiaceae), a new subspecies from the eastern USA. PhytoKeys 262: 31–44. https://doi.org/10.3897/phytokeys.262.165533

**DOI:** 10.3897/phytokeys.280.216670

**Published:** 2026-10-12

**Authors:** Jordan S. Metzgar

**Affiliations:** 1 Massey Herbarium (VPI), Department of Biological Sciences, Virginia Polytechnic Institute and State University, Blacksburg, Virginia, 24061, USA Massey Herbarium (VPI), Department of Biological Sciences, Virginia Polytechnic Institute and State University Blacksburg United States of America

The legend describing the illustrated morphological terminology of *Lygodium palmatum* (Bernh.) Sw. subspecies (Fig. 2) in my recent publication (Metzgar 2025) contained a typographic error. The subspecific epithets were mistakenly swapped in the legend. A corrected legend is provided below.

**Figure 2.**
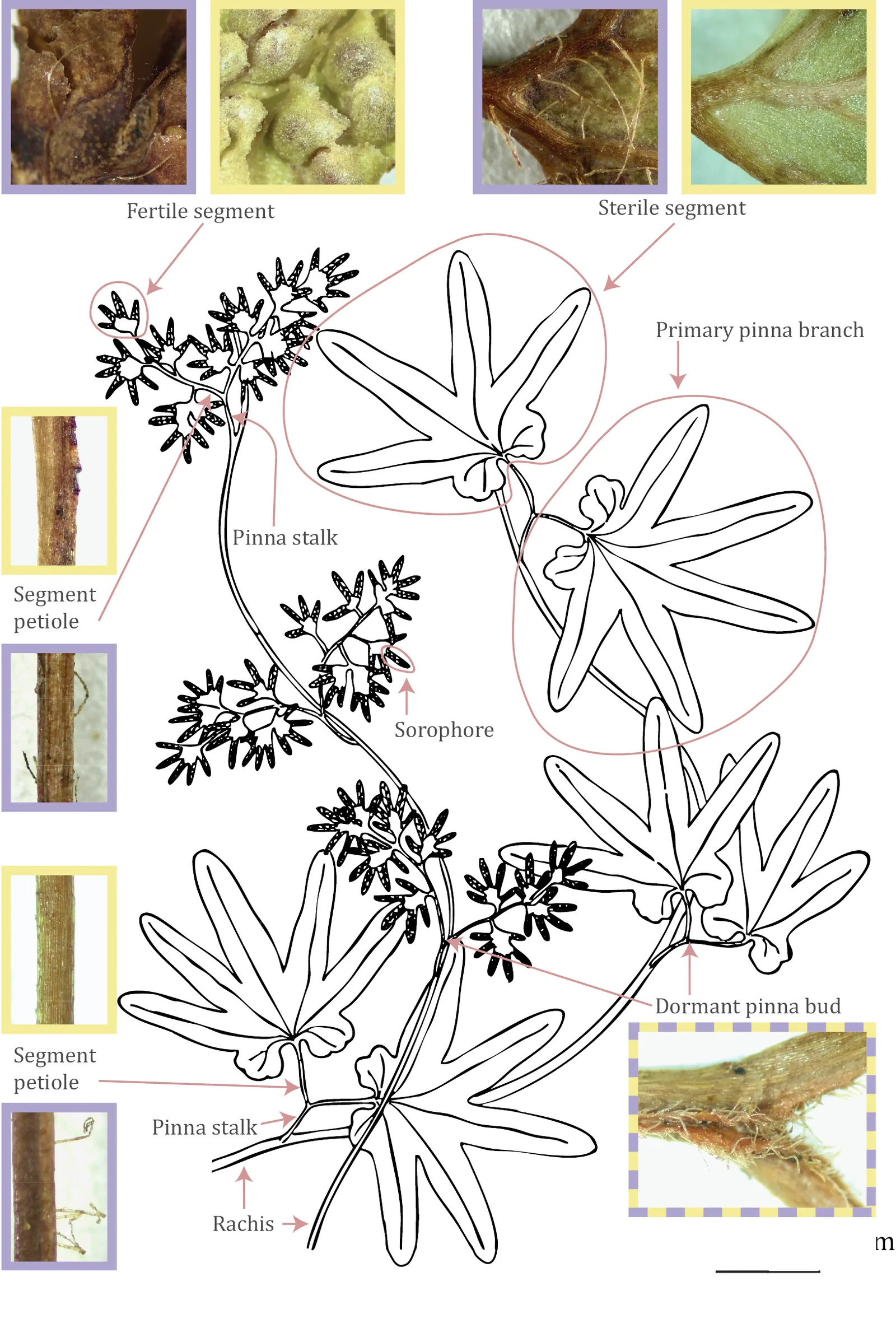
Illustrated morphological terminology. Both subspecies of *Lygodium palmatum* have twining fronds with complex dissection patterns. The main identification characters are illustrated with photomicrographs taken at 20× magnification, with the character state for Lygodium palmatum subsp. palmatum outlined in light purple and the character state for Lygodium palmatum subsp. puskariorum outlined in yellow. The dormant pinna bud character is invariant between subspecies and is outlined in both light purple and yellow. All photomicrographs for L. palmatum subsp. palmatum are from a single representative specimen (Flentje 7; VPI 112295). All photomicrographs for L. palmatum subsp. puskariorum are from one representative Massachusetts specimen (Ahles 78547; VPI 57060). The line drawing is modified from Ruth Sinclair George’s illustration (Small 1938).

## Additional information

### Artificial Intelligence (AI) use

The authors accept full responsibility for the content of the manuscript, including the disclosure of any use of AI.

No AI tools were used in the preparation of this manuscript.

### Author ORCIDs

J.S. Metzgar https://orcid.org/0000-0002-4641-9929

### Data availability

All of the data that support the findings of this study are available in the main text.
